# Musicians and non-musicians show different preference profiles for single chords of varying harmonic complexity

**DOI:** 10.1371/journal.pone.0281057

**Published:** 2023-02-02

**Authors:** Maria A. G. Witek, Tomas Matthews, Rebeka Bodak, Marta W. Blausz, Virginia Penhune, Peter Vuust

**Affiliations:** 1 Department of Music, School of Languages, Cultures, Art History and Music, University of Birmingham, Birmingham, United Kingdom; 2 Center for Music in the Brain, Aarhus University and Royal Academy of Music, Aarhus, Denmark; 3 Department of Psychology, University of Southern Denmark, Odense, Denmark; 4 Department of Psychology, Concordia University, Montreal, Canada; Northeastern University, UNITED STATES

## Abstract

The inverted U hypothesis in music predicts that listeners prefer intermediate levels of complexity. However, the shape of the liking response to harmonic complexity and the effect of musicianship remains unclear. Here, we tested whether the relationship between liking and harmonic complexity in single chords shows an inverted U shape and whether this U shape is different for musicians and non-musicians. We recorded these groups’ liking ratings for four levels of harmonic complexity, indexed by their level of acoustic roughness, as well as several measures of inter-individual difference. Results showed that there is an inverted U-shaped relationship between harmonic complexity and liking in both musicians and non-musicians, but that the shape of the U is different for the two groups. Non-musicians’ U is more left-skewed, with peak liking for low harmonic complexity, while musicians’ U is more right-skewed, with highest ratings for medium and low complexity. Furthermore, musicians who showed greater liking for medium compared to low complexity chords reported higher levels of active musical engagement and higher levels of openness to experience. This suggests that a combination of practical musical experience and personality is reflected in musicians’ inverted U-shaped preference response to harmonic complexity in chords.

## Introduction

How much we like certain music is explained by responses to its acoustic and structural properties as well as our engagement in playing and listening, and differences in personality, experience and culture [1]. Broadly speaking, listeners tend to prefer moderately complex music–that is, music that is neither too simple nor too complex [2]. For example, preference ratings tend to be highest for intermediate levels of harmonic complexity [3]. Musical training has been shown to influence the degree of complexity that listeners find most appealing [4–6]. However, which aspects of musicianship might contribute to shifting this preference have been little explored.

The inverted U hypothesis is a general principle that has been proposed to explain the preference for intermediate complexity in music [2]. Broadly speaking, it predicts that intermediate levels of complexity are generally liked better than low or high levels, producing a negative quadratic function on preference, appearing graphically like an upside-down (inverted) U. The hypothesis is based on Berlyne’s [7] psychobiological theory, in which aesthetic preference for complexity in art is driven by arousal. It has received extensive empirical support, especially in music research. For example, listeners prefer medium complexity in popular music [8], classical [9, 10], Caribbean, African, Indian and Papa New Guinean music [11] and jazz [12, 13]. The inverted U-shaped response has been shown for a variety of different musical properties, including rhythmic syncopation [14–16], tonal tension between chords in a harmonic progression [17] and information content and entropy [17, 18].

The harmonic complexity of single chords in music is an important contributor to preference. Each chord is a combination of notes, which can have a more or less complex relation with each other. This ‘vertical’ dimension of harmonic complexity (as distinguished from ‘horizontal’ complexity arising from the transition between chords in a harmonic progression) reflects the level of consonance or dissonance in the chord, which is widely agreed to have a strong relationship to preference and pleasantness [see 19, for a review] at least for Western listeners [20].

Consonance and dissonance can be modelled in different ways. Helmholtz [21] modelled dissonance as roughness, which is the perceptual dimension of the psychoacoustic phenomenon of beating. When a sound consists of two or more frequencies that are too close together, the hair cells inside the cochlea are unable to differentiate between them, becoming excited by both, and we hear beats as a result–a single frequency with periodically rising and falling intensity [22]. As the frequency difference increases, the sound begins to acquire a roughness or harshness to it, eventually leading to the perception of two distinct sounds. A recent study found that roughness is the strongest predictor of the prevalence of chords in classical, pop and jazz music [19]. This suggests that roughness is a good index of consonance and harmonic complexity in chords within Western tonal music.

There is evidence that listeners prefer medium degrees of harmonic complexity, consonance and roughness in single chords, consistent with the inverted U hypothesis [3, 23]. Lahdelma and Eerola [3] showed that minor^7^, major^9^ and minor^9^ chords were liked the most, and these chords have intermediate levels of roughness compared to other chords used in the study, e.g. major, minor (low roughness), and dominant^9^ and hexatonic chords (high roughness). Studies by Matthews et al. tested the relation between harmonic and rhythmic complexity on pleasure ratings in the context of groove (syncopated single chord patterns), focusing on the full [16] or the right half of the inverted U [24]. Both studies showed that medium harmonic complexity boosted pleasure ratings for patterns with medium compared to high rhythmic complexity, demonstrating an interaction between the two complexity measures. Interestingly, in the 2019 study, they found an inverted U-shaped effect for rhythm, but not for harmony, which showed no significant difference between low and medium complexity. They suggest that the rhythmic complexity may have been the primary driver of ratings here, due to the groove context, possibly masking any secondary effects caused by the harmonic complexity. Alternatively, insufficient levels of harmonic complexity may have been tested (three), preventing the identification of the full inverted U-shaped effect.

As discussed above, inter-individual differences also influence what music we like or dislike. For example, there is evidence of some cultural variation in the preference for consonance [4, 25], casting doubt on a long-held belief that preference for consonance is universal. With regard to musical training, a number of studies show that the more listeners have experience playing and engaging with music, the stronger the relationship between consonance and preference [4–6]. However, there is lack of clarity about the effect that musical training has on the inverted U-shaped relation between consonance and preference. For complexity as a subjectively rated property of music, North and Hargreaves [8] found that moderately trained musicians preferred higher levels of complexity than non-musicians, but we do not know if this is reflected in preferences for harmonic complexity, specifically. Lahdelma & Eerola [3] found no effect of musical training on the rating of chords in their study, while Popescu et al. [26] showed that the ability to dissociate roughness from pleasantness was positively correlated with musical sophistication. Matthews et al. [16] found that, while finding no clear inverted U-shaped effect of harmonic complexity, increased musical training led to decreased pleasure ratings for low complexity chords, pulling the relationship towards an inverted U.

In other words, if there are differences between musicians and non-musicians in liking for harmonically complex chords, it is unclear how this difference is manifested. On the one hand, there may be overall differences, with musicians showing higher liking overall, due to their overall greater familiarity with and knowledge of music in general. On the other hand, the difference may be in the shape of the inverted U, with musicians’ vertex at higher levels of complexity, indicating a greater aesthetic tolerance for harmonic complexity than non-musicians. The shape could also be sharper for musicians, showing greater sensitivity to harmonic complexity in preference response. Finally, it is unclear what aspects of musicianship might be associated with these differences. That is, is it simply the years spent practicing and learning to play their instrument, or might there be other inter-individual differences between musicians and non-musicians that affect their liking responses to harmonically complex chords?

Musicians have been shown to differ from non-musicians in a number of ways. Musical training is associated with stronger pleasurable experiences of music [27], reflected partly in their tendency to seek out music and be emotionally moved by music [28]. Musicians’ genre preferences also differ from those of non-musicians, with increased preference for classical, blues, folk and jazz music in particular [29]. Since different genres will employ different types of harmonic complexity [26], this may affect harmonic complexity preference. There is also evidence for personality differences among musicians and non-musicians [30–32]. In fact, musical competence is predicted by a personality measure known as openness to experience [33]. Openness also affects the inverted U-shaped relationship between musical preference and amount of exposure to music, presenting it as more right-leaning, suggesting greater tolerance for repeated exposures [34]. It is also associated with greater preference for classical, blues, folk and jazz music [35]. Finally, it may be that musicians’ superior musical perception abilities affect their preferences for harmonically complex chords. We know, for example, that musicians are better at discriminating between different musical patterns, and that these discrimination abilities can clearly differentiate between different levels of professionalism and training among musicians [36].

In this study, we investigated whether there is an inverted U-shaped relationship between liking ratings and harmonic complexity in single chords as measured by their acoustic roughness. We compared musicians and non-musicians, and further investigated what aspect of musicianship–levels of training, engagement, genre preference, types of personality or other inter-individual differences–contribute to any difference between these groups. We measured musical sophistication using the Goldsmith Musical Sophistication Index, focusing on two dimensions; musical training and active engagement [37]. To explore other individual differences among the two groups, we tested their tendencies for hedonic responses to music [28], their genre listening preferences [35], personalities [38, 39] and music perception abilities with a melodic discrimination task [36].

## Materials and methods

### Participants

Table 1 reports demographic data. We recruited 51 participants for the study, two of whom were excluded due to missing data, resulting in a final 49 participants aged between 20 and 49 years old. Twenty-five participants were musicians, with more than 8 years of musical training and currently practicing music. Twenty-four participants were non-musicians, with less than one year of musical training and not currently practicing music. Except for one Polish and one Norwegian, all participants were Danish. The musicians played a variety of genres, approximately evenly split across classical, pop, rock and jazz, with a minority playing electronic music, folk and blues.

**Table 1:** Demographic information, and independent samples t-tests comparing musicians and non-musicians on the Goldsmiths’ Musical Sophistication Index (GoldMSI), The Barcellona Music Reward Questionnaire (BMRQ), the Short Test for Music Preference (STOMP), Big Five Inventory (BFI) and Musical Ear Test (MET) scores. P-values are FDR corrected, *** p < .001, ** p < .01, * p < .05.

	Musicians	Non-Musicians	T-test	p-value
**N (female/male)**	25 (15/12)	24 (12/12)		
**Age (SD)**	24.29 (3.51)	23.20 (2.53)	1.22	0.311
**Years Musical Training**	13.21 (4.27)	-		
**Hours of Weekly Practice**	9.75 (6.61)	-		
**MET Melody**	43.96 (3.79)	34.6 (4.98)	7.41	< .001***
**GoldMSI-Active Engagement**	45.54 (6.99)	34.84 (8.59)	3.90	< .001***
**GoldMSI-Musical Training**	41.67 (3.74)	11.16 (3.79)	28.35	< .001***
**BMRQ-Music Seeking**	53.37 (8.04)	54.68 (8.04)	-0.57	0.640
**BMRQ-Emotional Evocation**	55.92 (5.86)	45.2 (10.64)	4.39	< .001***
**BMRQ-Mood Regulation**	46.96 (9.18)	45.76 (8.08)	0.48	0.665
**BMRQ-Sensorimotor**	48.96 (8.30)	45.8 (10.75)	1.15	.323
**BMRQ-Social**	62 (8.10)	47.48 (8.60)	6.09	< .001***
**BMRQ-Music Reward**	54.12 (7.08)	46.34 (8.78)	3.41	< .01**
**STOMP-classical, blues, folk and jazz**	20.45 (3.73)	15.65 (4.00)	4.36	< .001***
**STOMP-Rock, Alternative and Heavy Metal**	13.46 (3.02)	11.68 (3.79)	1.82	.130
**STOMP-Country, Soundtrack, Religious and Pop**	17.79 (2.78)	15.24 (3.87)	2.66	.02*
**STOMP-Rap/Hip-hop, Soul/Funk and Electronic Dance Music**	12.92 (3.69)	14.96 (3.56)	-1.97	.104
**BFI-Extrovert**	112.62 (16.21)	116.32 (20.44)	-0.70	.577
**BFI-Neurotic**	98.21 (14.98)	88.28 (19.01)	2.03	.101
**BFI-Agreeable**	110.04 (14.43)	108.88 (17.87)	0.25	.803
**BFI-Open**	115 (15.09)	109.52 (13.98)	1.32	.284
**BFI-Conscientious**	105.46 (14.82)	110.88 (13.60)	-1.33	.284

Informed written consent was obtained for all participants. The study was conducted through the Centre for Music in the Brain at Aarhus University, therefore, ethics were governed by the Central Denmark Region Committees on Health Research Ethics. According to their Act on Research Ethics Review of Health Research Projects (Act 593 of 14 July 2011, section 14.1), only health research studies shall be notified to the Committees. Our study is not considered a health research study (section 14.2) and therefore did not require ethical approval.

### Stimuli

**Fig 1:**
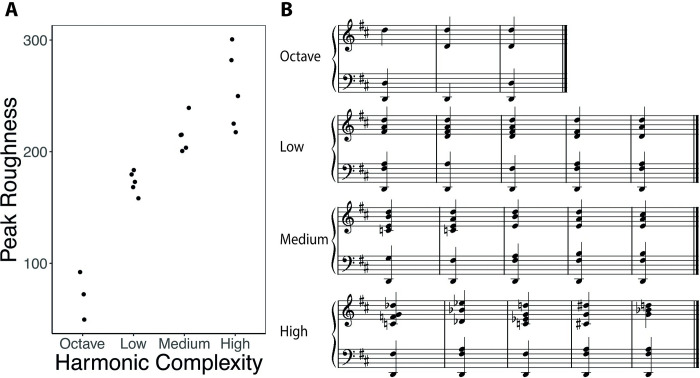
**A**. Peak acoustic roughness of harmonic complexity conditions. **B**. Notational transcriptions of chords used for each level of harmonic complexity. For the octave, the final chord was repeated twice to obtain five (see methods).

Participants heard a set of 20 individual piano chords which were generated using Cubase Pro version 9.0.30 (Steinberg Media Technologies). Chords were created according to four levels of harmonic complexity; octave, low, medium and high. To stay consistent with and extend our previous research, we use the same chords for low, medium and high as in Matthews et al 2019 and 2020. These chords were chosen based on understanding of harmonic complexity from music theory [40–42]. There were five chords at each level of complexity. All chords were in D major key spanning four octaves (D2 to #D5; see Fig 1B), comprising six tones, except the octave which only comprised three. Due to the limited number of chord configurations possible for the octave within the pitch range (max three), we duplicated one octave chord to obtain five. Low complexity chords consisted of the D major triad and four inversions. Medium complexity chords consisted of four-note major chords with extensions. High complexity chords included a flat ninth interval between chord note and extension which is considered highly dissonant, when not specifically occurring as flat 9^th^ on major 7^th^ chord, according to contemporary harmonic theory [40–42].

In addition, we measured harmonic complexity according to acoustic roughness, using the Sethares method in the MIR toolbox [43, 44]. Fig 1A reports the peak roughness measures for each harmonic complexity category. While there is mostly a clear separation of roughness between the four levels, there is one chord in the medium level that overlaps with roughness for high complexity. This may be a reflection of the nature of roughness, which is only one of several contributors to dissonance [19, 45].

### Other measures

To explore the musical and personal characteristics of musicians and non-musicians, we recorded a number of background measures. We used the Goldsmiths Musical Sophistication Index v.1.0 (GoldMSI, 39) to record musical engagement, which is a more general measure of musical interest outside of any formal training. It represents how much time and money people spend on music activates (e.g., listening, searching on the internet, keeping track of new music). We also used the GoldMSI measure of training, which reflects years of training, average time spent practicing instruments (hours per day) and whether they consider themselves and are considered by others as a musician.

The Barcelona Music Reward Questionnaire (BMRQ) [28] was translated to Danish and used to record participants’ sensitivity to rewarding experience from music, according to five factors; music seeking, emotional evocation, mood regulation, social and sensorimotor reward. There is also an aggregate factor–music reward–representing average scores across these factors. The measure includes 20 items rated from 1 (Totally Agree Completely) to 5 (Totally Disagree).

Participants’ genre listening preferences were recorded using a Danish translation of the Short Test of Music Preferences (STOMP), which consists of 14 items, rated from 1 (Do not like at all) to 7 (Like a lot). The study on which the STOMP is based [35] grouped genre preferences into categories based on factor analysis, and named the factors according to the listening functions they thought most important for those genres; reflective and complex (including classical, blues, folk and jazz), intense and rebellious (including rock, alternative and heavy metal), upbeat and conventional (including country, sound tracks, religious and pop music) and energetic and rhythmic (including rap/hip-hop, soul/funk and electronic dance music). However, we disagree with the implications that these listening function categories have for the understanding of the experience of these genres. For example, electronic dance music can be highly rhythmically complex [46] and intense [47], pop music can be experienced with a reflective mood [48], and jazz can be upbeat and conventional [49]. Therefore, we use the genre names instead of the listening functions to refer to the different factors.

The ‘Big Five Inventory’ (BFI) of personality traits were measured using the shortened version of the International Personality Item Pool of the NEO PI-R [IPIP-NEO, 50], which indexes individual levels of openness, conscientiousness, extroversion, agreeableness and neuroticism. We used a Danish version, which includes 163 items [51] scored on a Likert scale from 1 (Strongly Disagree) to 5 (Strongly Agree).

Finally, we employed the melody part of the Musical Ear Test (MET)–a discrimination task that asks participants to listen to 52 pairs of melodies and indicate on a paper sheet whether they are the same or not the same [36]. The MET indexes participants’ abilities to differentiate tonal relationships in a melody, and as such may affect preferences for tonal relationships in chords (i.e., harmonic complexity).

Means for musicians and non-musicians on the GoldMSI, BMRQ, STOMP, BFI and MET were compared using independent t-tests corrected with the False Discovery Rate (FDR) method (see Table 1). These tests showed that musicians scored significantly higher on GoldMSI-active engagement, GoldMSI-music training, STOMP-classical, blues, folk and jazz, STOMP-country, soundtrack, religious and pop, BMRQ-emotional evocation, BMRQ-Social, BMRQ-music reward and the MET-melody test.

### Procedure

Participants completed the tasks in groups of 1–5 in a computer room at the Royal Conservatory of Music, Aarhus, Denmark, each on individual computers using individual headphones. Upon arrival, participants gave informed consent. Then, they completed a brief demographics questionnaire, followed by a short training session for the chord listening experiment, then the experiment proper. Participants heard each chord twice per trial, with a two-second silence between chords and preceded by one of three versions of a masking sequence. There was a two-second silence between the mask and the first chord in the trial. The purpose of the mask was to remove any perception of harmonic progression between the chords. The masks were made up of sixteen notes lasting 200 ms, with 200 ms inter-onset-intervals, using the same grand piano instrument as for the chords themselves. One version of the note sequence consisted of the pitches B-Eb-C#-D-C-Bb-G#-A-C-Bb-D-E-B-G#-A-C#, in that order, and suggested no tonal or harmonic center. The other two versions consisted of these pitches transposed up by a major 2nd and major 5th, respectively. The chords and masks were fully randomized across participants. After the second repetition of each chord, participants had 7 seconds to rate how much they liked the chord on a Likert scale from 1 (Not at all) to 5 (Very much). Afterwards, they completed the MET, followed by the GoldMSI, BMRQ, STOMP and BFI, in that order. The experiment took approximately 30 mins in total.

### Analysis

We used linear mixed effects regression with a hierarchical approach to test effects of harmonic complexity on chord liking and interactions with musician group. The complexity and group fixed effects were coded using effects coding (complexity: octave = -.5, low = -.25, medium = .25, high = 0.5. Group: non-musician = -.5, musician = .5). Polynomial contrasts (linear and quadratic) were specified for harmonic complexity. We determined the random structure for our models by following the procedures laid out by Bates et al. [52]. We started off with the maximal random structure, including by-participant and by-chord random quadratic slopes and intercepts, and then reduced it to the optimal structure that could be supported by the data. The resulting random structure included a by-participant random quadratic slope and intercept and by-chord random intercept.

We then added the fixed factors incrementally, including first the polynomial term for harmonic complexity, then the group factor of musicianship, and finally the interaction, assessing model fit using the likelihood ratio test [53]. From this model, we used emmeans to calculate estimates of mean differences and post-hoc contrasts corrected for multiple comparisons using the multivatiate t-method [54]. Confidence intervals and p-values were calculated using degrees of freedom approximated with the Satterwaithe method. Model residuals were homoscedastic and normally distributed.

Due to the high number of measures of individual differences, each also including several subscales (Table 1), these were not included in the main analysis, but instead addressed in post-hoc exploratory analyses aimed at further elucidating the main findings from the linear mixed effects model.

## Results

**Fig 2:**
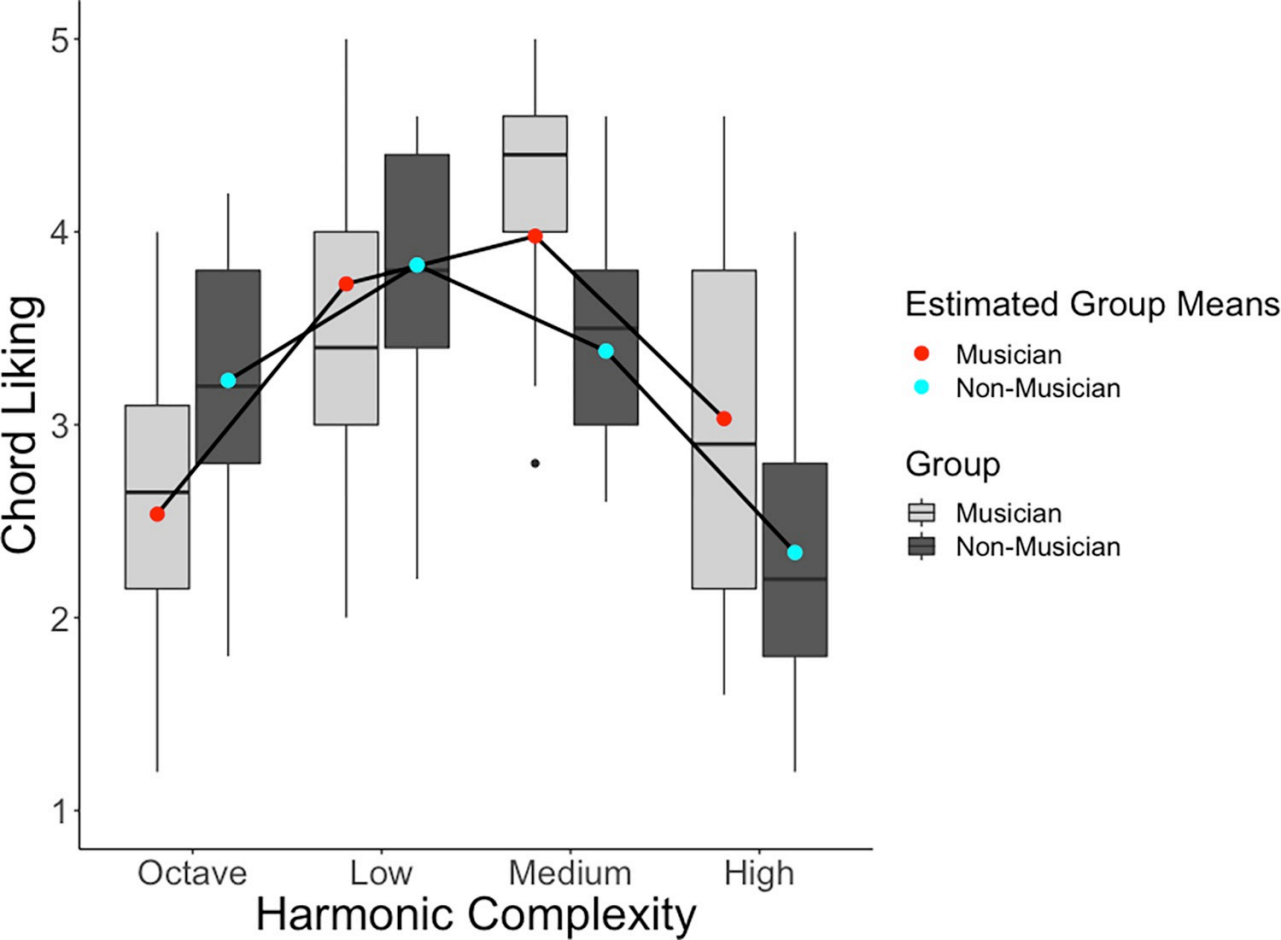
Effects of harmonic complexity and musicianship on chord liking ratings. Tukey-style boxplots represent the raw data, with the horizontal lines representing the median. Red and cyan dots and black connecting lines represent estimated means from the linear mixed effects model.

Our linear mixed effects model showed that model fit was significantly improved by adding harmonic complexity (χ^2^(2) = 63.05, p < .001), group (χ^2^(2) = 4.31, p = .038) and the complexity-by-group interaction (χ^2^(2) = 19.70, p < .001). Model coefficients are reported in Table 2 with p-values. The quadratic term for harmonic complexity was significant, with a negative sign, suggesting an inverted U-shaped effect on liking ratings. There was also a significant interaction with group, but only with the linear term for harmonic complexity. We investigated this further in post-hoc contrasts, reported in Table 3. This showed that both groups exhibited an inverted U-shaped relationship between complexity and liking, but with different vertices and start and end positions (Fig 2); Non-musicians liked low complexity chords significantly more than the octave, medium and high complexity chords. Medium was significantly liked more than high, but not significantly more than the octave. The octave was liked significantly more than the high complexity chords. For musicians, medium complexity chords were liked significantly more than octave and high complexity chords. Low complexity chords were liked significantly more than the octave and high complexity chords. There was no significant difference between low and medium, or octave and high for musicians. Comparing musicians with non-musicians at each level of complexity showed significant increased liking for non-musicians at the octave, and for musicians at medium and high complexity chords.

**Table 2:** Model coefficients and test statistics, testing effects of musicianship group, linear and polynomial effects of harmonic complexity, and interaction between group and harmonic complexity. ** indicates significance at p <. 001.

	Estimate	SE	df	t	*p*
**Harmonic Complexity (linear)**	-2.455	1.93	49.03	-1.268	ns
**Harmonic Complexity (quadratic)**	-14.800	1.28	48.96	-11.567	< .001**
**Musicianship Group**	0.012	0.12	48.92	1.010	ns
Harmonic Complexity (linear)*Group	17.163	3.87	49.03	4.343	< .001**
Harmonic Complexity (quadratic)*Group	-3.892	2.56	48.96	-1.521	ns

**Table 3:** Post-hoc contrast estimates, comparing each level of harmonic complexity within each musicianship group, and each musicianship group within each level of harmonic complexity. * indicates p < .05.

		Estimate	SE	df	CI
Group	Harmonic Complexity				
Non-Musician	Octave>Low	-0.590	0.125	45.977	[-0.945, -0.251]*
	Octave>Medium	-0.181	0.192	46.026	[-0.693, 0.388]
	Octave>High	0.819	0.213	46.009	[0.259, 1.525]*
	Low>Medium	0.410	0.107	46.009	[0.129, 0.762]*
	Low>High	1.409	0.203	46.001	[0.876, 2.104]*
	Medium>High	1.000	0.130	45.985	[0.659, 1.430]*
Musician	Octave>Low	-1.201	0.127	46.029	[-1.548, -0.840]*
	Octave>Medium	-1.411	0.196	46.100	[-1.993, -0.889]*
	Octave>High	-0.420	0.218	46.053	[-1.141, 0.151]
	Low>Medium	-0.210	0.109	46.053	[-0.571, 0.076]
	Low>High	0.782	0.207	45.999	[0.071, 1.326]*
	Medium>High	0.992	0.133	45.978	[0.553, 1.340]*
Non-Musician>Musician	Octave	0.760	0.241	49.345	[0.017, 1.369]*
	Low	0.149	0.152	51.664	[-0.337, 0.533]
	Medium	-0.470	0.130	48.525	[-0.962, -0.229]*
	High	-0.479	0.227	50.708	[-1.375, -0.013]*

**Fig 3:**
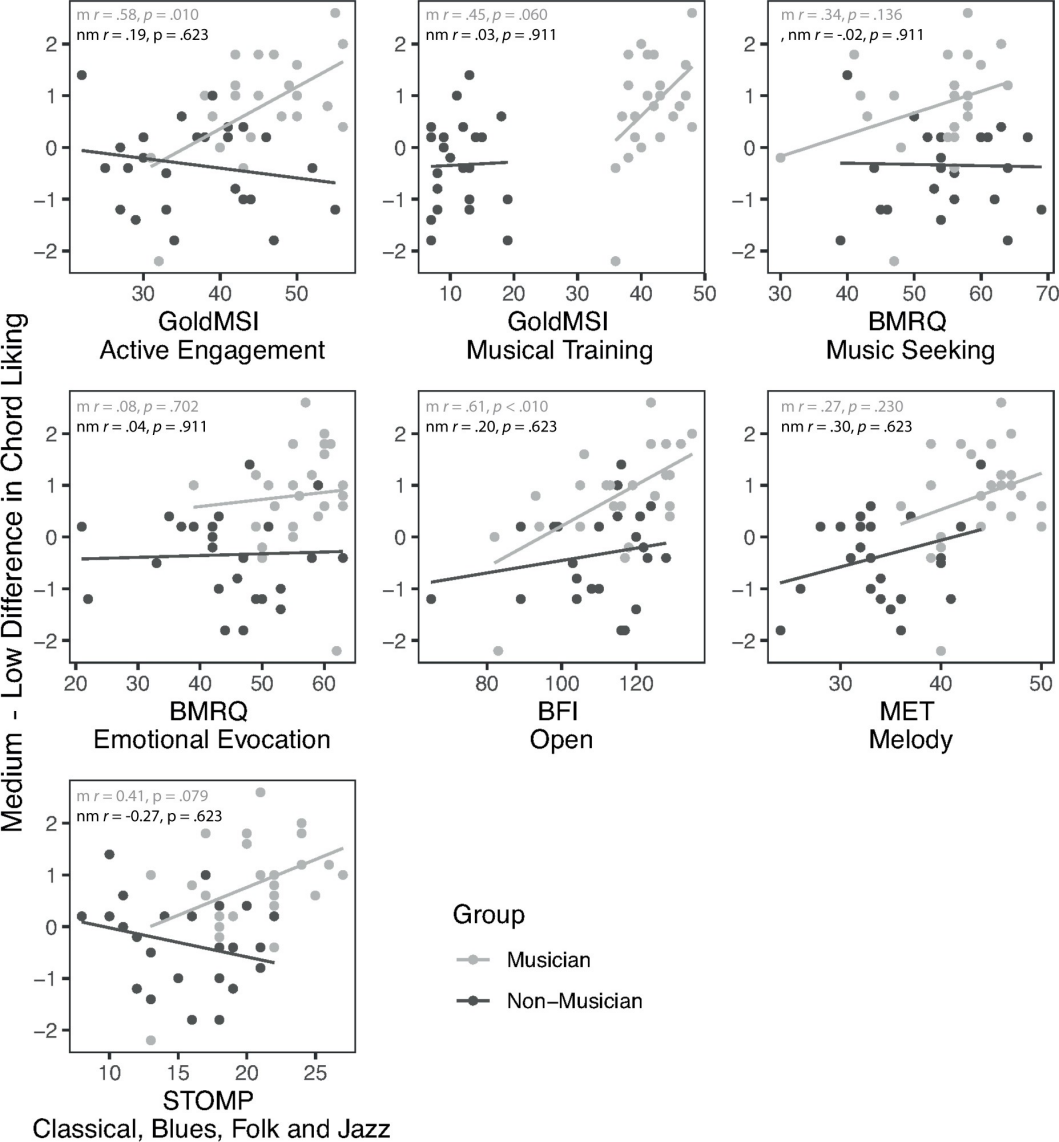
Scatterplots and Pearson’s correlation coefficients (m = musician, nm = non-musician). The y axis represents the difference in ratings between medium—low harmonic complexity. The x axes represent scores on individual difference measures. P values are corrected for multiple comparisons using the FDR method, with significance at p < .05.

To further investigate the difference in the U-shape and peak liking for the two groups, we calculated the difference between ratings for medium and low complexity chords for both groups. We did not investigate medium vs high, since the estimated means plotted in Fig 2 indicated that the two groups exhibited a similar pattern of preference ratings between these two complexity levels (i.e., the lines are parallel). We calculated the difference scores by subtracting low ratings from medium ratings for each subject averaged across items. We then plotted these difference scores for each group against the various background and individual difference measures (Table 1). The plots can be found in Fig 3. We chose a selection of measures for this analysis, based on previous research. Both GoldMSI measures were included, to investigate whether formal training or more general musical engagement affects the response. From the STOMP, we tested the measure of preference for classical, blues, folk and jazz, since it has been shown to increase for musicians [29]. From the BMRQ, we included ‘Music Seeking’ and ‘Emotional Evocation’, since these have been shown to differentiate musicians and non-musicians in the past [28]. Since openness to experience is known to be associated with both musicianship [33] and changes to the U-shaped preference response [34], we included this measure from the BFI. Finally, we included the Melody MET test, to see whether melodic perception abilities were associated with the change in the U-shape between musicians and non-musicians. We also calculated correlations between the difference scores and these variables, separately for each group using Pearson’s *r* (values added to plot in Fig 3).

The plots show that musicians’ difference scores tended to be positive, while non-musicians tended to be negative, reflecting the main finding that musicians’ liking is higher for medium, while non-musicians’ liking is higher for low harmonic complexity. After correcting for multiple comparisons using the FDR method, there were two significant and two close-to-significant correlations in the musician group and none in the non-musician group. We interpret the significant correlations in relation to the scatterplots in Fig 3. There was a significant large positive correlation between difference score and GoldMSI-Active Engagement. A similar pattern was seen for GoldMSI-Musical Training, with a medium size effect, but here the p-value was only close-to-significant following correction. There was also a significant large positive correlation for BFI Openness and a close-to-significant medium positive correlation for STOMP-classical, blues, folk and jazz. These correlations suggest that the higher musicians score on certain individual difference measures, the greater the increase in liking for medium over low harmonic complexity chords.

## Discussion

We show that there is an inverted U-shaped relationship between harmonic complexity in single chords, indexed by their acoustic roughness, and liking ratings in both musicians and non-musicians. This supports previous studies [3, 23], and further shows the effect for a different set of chords, suggesting the effect is not limited to the harmonic note combinations used in previous studies. In addition, we find that the shape of the inverted U differed for musicians and non-musicians. For musicians, liking ratings peaked for medium complexity chords and were lowest for the octave, whereas for non-musicians liking ratings peaked for low complexity chords and were lowest for high complexity chords. Furthermore, for musicians only, higher levels of active engagement with music and greater openness to experience were related to higher ratings for medium compared to low complexity chords. This suggests that the differences in the inverted U-shaped relationship between liking and harmonic complexity in musicians is associated with a mixture of musical experience and personality.

Liking responses to music complexity have previously been found to differ depending on a listeners’ level of musical training [9, 55–57], although not all studies have shown this effect [3]. There is a longstanding hypothesis that more expertise makes a person more tolerant of complexity [58] based on the idea that the greater exposure to and explicit knowledge of musical structures that comes with practicing and performing music increases the ability to fit more complex structures into an acceptable musical context. Our results align with this hypothesis. Specifically, non-musicians preferred low harmonic complexity, while musicians showed comparable preference for both low and medium. Furthermore, musicians’ ratings were significantly higher for medium and high complexity compared to non-musicians. We also found differences in the least preferred level of harmonic complexity; musicians least preferred low, while non-musicians least preferred high. Together, these results show that non-musicians’ preferences are skewed towards lower complexity, while musicians’ preferences are skewed towards higher complexity. These results are also in line with North and Hargreaves [8], who showed that the vertex of the inverted U was slightly more right-skewed for moderately trained musicians compared to non-musicians, when rating overall complexity in popular music. Our results suggest that musicians’ preference for more complex harmonies may be a building block for their preference for overall greater complexity in real music.

However, not all previous research aligns with this direction of difference for musicians’ and non-musicians’ complexity preferences, suggesting that the factors affecting preference can be complex and context-sensitive. For example, Orr and Ohlsson [59] found that the inverted U was only clearly present for listeners with no musical training, while professional and amateur musicians showed a much weaker or non-existent effect. Results are also mixed for studies investigating harmonic complexity and consonance, specifically. Lahdelma and Eerola [3] found no effect of musical training when assessing their inverted U-shaped relation between harmonic complexity in chords and preference, although they did not compare distinct musicianship groups as we do in the current study. Furthermore, it appears that expertise can augment the dislike for dissonant chords, as shown in Bigand et al [5]. This was supported by Dellacherie et al [6], where musicians rated dissonant piano music as more unpleasant than non-musicians. It may be that the nature of the difference between musicians’ and non-musicians’ inverted U-response to musical complexity depends strongly on the type of stimuli used, the way that complexity is measured and how the musicianship groups are defined [2].

In order to better understand our group differences, we focused in on the difference between ratings for low and medium harmonic complexity. We found that, for musicians, this difference score correlated with levels of active engagement with music recorded with the GoldMSI tool [37], suggesting that the more actively engaged musicians are with music, the more they prefer medium over low complexity. The same relationship was found for musical training, although below the corrected threshold. Overall, the results are in line with those by Matthews et al. [16], who found that increased musical training led to decreased pleasure ratings for low harmonic complexity. As musicians progress in their training and explore their musical interests, they gain more detailed knowledge of musical structures of varying complexity, making them better able to process and thus appreciate higher levels of complexity. Interestingly, the association between the difference score and active musical engagement was only present for musicians and not for non-musicians. It may be that there is a threshold above which active engagement with music begins to be reflected in listening preferences for complex harmonic chords. Our results are also interesting to consider in relation to those by Popescu et al. [26]. There, a negative linear relationship was found between rated roughness and pleasantness, but the higher their participants scored on the overall GoldMSI measure, the more they were able to dissociate roughness ratings from pleasantness, suggesting deviations from the linear effect. It may be that this ability to dissociate roughness from pleasantness explains why more trained and more actively engaged musicians have an inverted U-shaped response to harmonic complexity that is less left-skewed, i.e., less negatively linear overall.

The medium minus low difference scores also correlated with musicians’ openness to experience, measured using the ‘Big Five Inventory’ for personality. In other words, the more musicians are open to experience, the more the vertex on the inverted U-shaped effect of harmonic complexity on liking is shifted from the left to the right, indicating higher aesthetic tolerance for harmonic complexity. Openness to experience as measured via the BFI is a composite of multiple dimensions [60], several of which have been associated with characteristics which could be linked to preferences for higher levels of musical complexity, such as ‘aesthetic sensitivity’, ‘preference for variety’, ‘intellectual curiosity’ and ‘challenging authority’. This is supported by previous studies showing associations between openness to experience and listeners’ preference for complex music in general [61] and changes in the shape of the inverted U in response to familiar music [34]. Musicians tend to score higher on openness to experience [31, 32, 62–64], although this overall difference compared to non-musicians was not found in the present study. Openness to experience has also been found to predict how much musicians practice [65], the propensity towards experiencing chills from music [66] and preferences for classical, blues, folk and jazz music, as measured by STOMP [35, 67, 68].

The preference for classical, blues, folk and jazz was also found to correlate with the difference score in our study, although below the corrected threshold. This trend aligns with previous research. Popescu et al. [26] found that the ability to dissociate roughness from pleasantness was strongest for jazz and classical chords, in that order. Furthermore, the more musically sophisticated their participants, the more able they were to dissociate roughness from pleasantness in classical and jazz chords. We also found that mean ratings for these genres were greater for musicians than for non-musicians (Table 1), supporting previous research [29]. If we speculate that those musicians who reported a preference for classical, blues, folk and jazz also prefer to play these genres, our results might suggest that playing certain genres makes musicians more likely to report preference for higher complexity chords (please note that we were unable to test effect of genres played due to the small group sizes in our study). It is also likely that much of the training that our musicians had undergone required them to practice these genres, especially classical and jazz (these are key genres taught at the Danish conservatoires from which our sample was recruited), leading to increased knowledge of and preference for these styles and a greater association with the right-leaning inverted U-shaped effect of harmonic complexity. Furthermore, we cannot exclude the possibility that socio-cultural conventions surrounding the appreciation of certain genres may lead to biases towards reporting preference for higher levels of complexity. In other words, the musicians in our study with higher preferences for classical, blues, folk and jazz genres might have rated higher complexity as more preferred partly because chords with higher complexity are valued in some of these genres (i.e., classical and jazz).

It seems clear that musical expertise, personality and preference for certain musical genres are all important in explaining what types of musical structures musicians like. Here, we show that these factors are associated with a shift in the inverted U-shaped effect of harmonic complexity, suggesting that the factors may explain musicians’ higher tolerance for structural complexity in music. Our results suggest that aesthetic preference in music is associated with inter-individual differences that reflect both learning and personality factors. Why these factors are important and how they interact with each other to affect preference for higher complexity chords, remains for future research to determine. It will also be important to disentangle the context-specific effects that may have led to inconsistent findings in musical training and complexity preference research, more broadly. Finally, we suggest future researchers extend or replicate these results using continuous measures of complexity, to provide a more fine-grained model of the shift in the inverted U-shaped relationship between harmonic complexity and preference.
